# Molecular etiopathology of naturally occurring reproductive diseases in female goats

**DOI:** 10.14202/vetworld.2017.964-972

**Published:** 2017-08-22

**Authors:** V. Beena, R. V. S. Pawaiya, K. Gururaj, D. D. Singh, A. K. Mishra, N. K. Gangwar, V. K. Gupta, R. Singh, A. K. Sharma, M. Karikalan, Ashok Kumar

**Affiliations:** 1 Division of Animal Health, ICAR-Central Institute for Research on Goats, Makhdoom, Farah, Mathura - 281 122, Uttar Pradesh, India; 2 Centre for Animal Disease Research and Diagnosis (CADRAD), ICAR-Indian Veterinary Research Institute, Izatnagar - 243 122, Uttar Pradesh, India; 3 Division of Pathology, ICAR-Indian Veterinary Research Institute, Izatnagar - 243 122, Uttar Pradesh, India

**Keywords:** *Brucella melitensis*, *Campylobacter* spp, *Chlamydophila* spp, *Corynebacterium ovis*, etiopathology, female genitalia, goat, ovary, reproductive diseases, uterus

## Abstract

**Aim:**

The aim of the present study was to investigate the molecular etiopathology of occurrence of reproductive diseases in female goats. Reproductive diseases in goats account for major economic losses to goat farmers in terms of valuable loss of offspring and animal productivity.

**Materials and Methods:**

A total of 660 female genitalia were examined for pathological conditions (macroscopic and microscopic lesions). The etiopathological study was carried out for the presence of pathogenic organisms such as *Brucella*, *Chlamydia*, and *Campylobacter* in the uterus and ovary. Based on the microscopic lesions, suspected samples were subjected to diagnostic polymerase chain reaction (PCR) for various etiological agents employing 16srRNA genus specific primers for *Campylobacter* and *Chlamydophila* and *OMP31* gene-based PCR for *Brucella melitensis* and nested PCR using *ITS-1* gene primers for *Toxoplasma gondii*. For *Brucella* suspected samples, immunohistochemistry (IHC) was also performed.

**Results:**

In studied female genitalia, 108 (16.30%) showed gross abnormalities with overall 23.32% occurrence of pathological conditions (macroscopic and microscopic lesions). Pathological involvement of the uterus was the highest 68 (62.96%), followed by the ovaries 27 (25%) and other organs. Major uterine condition observed was endometritis (5.60%). In uterine infections, 35 (5.30%) samples were found positive for *Campylobacter* spp., 12 (1.81%) samples for *B. melitensis*, and 3 (0.45%) samples were positive for *Chlamydophila* spp. Among the samples positive for *B. melitensis* by PCR, 3 were found positive by IHC also. *Corynebacterium ovis* was detected by PCR using specific primers in a case of hydrosalpinx. It was concluded that many pathological lesions in female genitalia of functional significance play a major role in infertility in goats.

**Conclusion:**

The present study concluded that many pathological lesions in female genitalia of functional significance play a major role in infertility in goats.

## Introduction

Goats are multipurpose animals, producing meat, milk, skin, and hair and contribute greatly to food, rural employment, and gross domestic product. Genital abnormalities play an important role in animal breeding either by causing infertility or sterility and thus inflict heavy economic losses [1]. Lesions affecting the uterus and ovary greatly contribute to infertility or sterility in goats and hence compromise enterprise profitability. Susceptibility for reproductive abnormalities increases with age of the animal [2].

The major reproductive disorders in goats are abortion, retention of placenta, and stillbirth [3]. Dunn [4] analyzed the diseases of the periparturient goat mainly as dystocia and metritis. Reproductive tract lesions reduce the feed conversion efficacy and thereby reduce milk production and pregnancy rate and forcing the owners to cull the animal [5,6]. According to a study in goats, the uterus exhibited the highest (14.6%) prevalence of genital lesions [7], whereas endometritis and ovarian cysts were the most common abnormalities in the slaughtered ewes [8]. The most common infections to cause abortion and infertility in ewes are chlamydia and toxoplasmosis [9].

There is scanty published information regarding etiopathology and prevalence of various abnormalities in Indian goats [10,11]. The pathological investigation of abattoir specimens provides a great deal of information on the types and prevalence of genital tract lesions and abnormalities due to infections. Information on occurrence of major infectious causes of diseases of female reproductive system of goats could help in proper diagnosis and suitable preventive and control measures. Moreover, slaughter of infected animals is of great public health concern. Therefore, the prevalence and the pathological lesions produced specifically by organisms of public health importance (*Brucella, Chlamydia, Campylobacter*, and *Toxoplasma*) were investigated in this study.

## Materials and Methods

### Ethical approval

The study was conducted after due approval from IAEC and CPCSEA.

### Collection of reproductive organs

Female reproductive organs were collected from 660 goats from different locations in the Uttar Pradesh, Haryana, and Delhi states. The organs were collected immediately after slaughter of goats and transported to the pathology laboratory of the institute for further studies. In the laboratory, detailed examination of reproductive organs was carried out, and gross pathological changes were recorded. The representative tissue samples were preserved in 10% neutral buffered formalin for histopathological and immunohistochemical studies and refrigerated for molecular and isolation studies.

### Histopathology

Formalin-fixed tissues were processed routinely and paraffin-embedded tissue sections of 4-5 µm thickness were obtained with the help of semiautomatic rotary microtome (Leica RM 2145, Germany). The sections were stained with hematoxylin and eosin following earlier described procedures [12], and the slides were examined under light microscope for histopathological changes.

### Immunohistochemistry (IHC)

IHC technique was employed on formalin-fixed paraffin-embedded tissue sections for the detection of *Brucella Melitensis* specific antigen in the suspected cases which were positive by polymerase chain reaction (PCR) technique [13]. About 5 µm thick sections were taken on 3-aminopropyl-triethoxysilane (Sigma-Aldrich, USA) adhesive coated slides. Antigen retrieval for OMP31 (outer membrane protein) was done by microwave irradiation of tissue sections in 10 mM sodium citrate buffer (pH 6) for 5 min. Thereafter, the slides were incubated in 3% H_2_O_2_ in absolute methanol to quench the endogenous peroxidase, and blocking was done by 5% bovine serum albumin to decrease background staining. Immunostaining for OMP31 was performed by standard avidin-biotin complex (ABC) method, following the manufacturer’s instruction provided along with the product. Rabbit polyclonal antibody pSer167 (Sigma-Aldrich, USA; Product No. E7905) was used as a specific primary antibody (1:200 dilution) against OMP31 *B. melitensis* antigen. The diluent (1% phosphate buffered saline) alone was used as a negative control. A positive control slide of tissue known to contain the *Brucella* antigen was also used. Biotinylated goat anti-rabbit immunoglobulin G was used as secondary antibody followed by incubation with extra-avidin peroxidase (Sigma-Aldrich, USA; Product No. E8386). Chromogen 3-amino-9-ethylcarbazole was used as substrate for which produced brick red positive color reaction. The sections were counterstained with Mayer’s hematoxylin, mounted with glycerol gelatin, and observed under light microscope.

### PCR

Tissue samples stored in deep freeze (−20°C) were used for DNA extraction using commercially available QIAamp DNA Mini Kit (Qiagen, USA; Cat No./ID 51304). Around 100 µl of DNA was extracted from each tissue sample (mainly uterus) and performed PCR for *B. melitensis* [14], *Toxoplasma gondii* [15], *Campylobacter* spp., and *Chlamydophila* spp. [16], using a pair of species-specific primers for *B. melitensis, T. gondii*, and genus-specific primers for *Chlamydia* spp. and *Campylobacter* spp. as well as *Corynebacterium* spp. from published primers [17]. For *Campylobacter* spp., primers were designed from the conservative 16srRNA sequence using BioEdit [18]. The amplified products were subjected to gel electrophoresis in 1.5% safe view gel stained Tris-acetate-ethylenediaminetetraacetic acid agarose and visualized under ultraviolet trans-illuminator. The primers used in the current study are given in Table 1.

**Table-1:** List of primers used for PCR amplification for various organisms.

Organisms	Name of primer	Primer sequence	Size of PCR products
*B. melitensis*	OMP-31	F: 5’-TGACAGACTTTTTCGCCGAA-3’	1.1 kb
		R: 5’-TATGGATTGCAGCACCGC-3R:
*Chlamydophila* spp.	16SrRNA	F: 5’-TGTCGTCAGCTCGTGTCGTG-3’	277 kb
		R: 5’-TCTACGATTACTAGCGATTCCG-3’
*Campylobacter* spp.	16SrRNA	F: 5’-TGTCGTCAGCTCGTGTCGTG-3’	276 kb
		R:5’-TCTACGATTACTAGCGATTCCG-3’
*T. gondii* (nested PCR)	Ex:NN1	F: 5’-CCTTTGAATCCCAAGCAAAACATGAG-3’	227 kb
	NN2	R: 5’GCGAGCCAAGACATCCATTGCTGA-3’	
	In:TgNP1	F: 5’GTGATAGTATCGAAAGGTAT-3’	
	TgNP2	R: 5’-ACTCTCTCTCAAATGTTCCT-3’	
*C. ovis*	16SrRNA	F: 5’-ACCGCACTTTAGTGTGTGTG-3’	815 kb
		R: 5’-TCTCTACGCCGATCTTGTAT-3’	

F=Forward primer, R=Reverse primer, Ex=External primer, In=Internal primers, *B. melitensis=Brucella* *melitensis*, *T. gondii=Toxoplasma gondii*, *C. ovis=Corynebacterium ovis,* PCR=Polymerase chain reaction

### Isolation and identification of *Brucella*

Tissue samples preserved at 4°C were used for isolation studies. For isolation of *Brucella*, a loopful of tissue scrapings were inoculated by spreading on plates of sterile *Brucella* agar with Hemin and Vitamin K1 (HiMedia, Mumbai) and incubated at 37°C for 48 h. Confirmation of the pure culture was done using biochemical tests (result not shown) and staining.

## Results

Intact female reproductive tracts were collected from 660 goats slaughtered in abattoirs at different locations in the Uttar Pradesh, Haryana, and Delhi states as well as from postmortem house of the institute. Of 660 female genitalia examined, 108 (16.30%) showed gross abnormalities, with overall 23.32% (154/660) occurrence of pathological conditions (macroscopic and microscopic lesions). Among 108 grossly abnormal genitalia, pathological involvement of the uterus was the highest 68 (62.96%) followed by ovaries (27; 25%), with the overall occurrence of 12.42% and 6.06%, respectively. Of uterine pathologies, endometritis (37/660; 5.60%) was the major condition observed.

During the study, DNA was extracted from 76 samples suspected for infections. Out of 76 samples selected, 35 (5.30%) samples were found to be positive for *Campylobacter* spp., 12 (1.81%) for *B. melitensis*, and 3 (0.45%) for *Chlamydia*. However, *T. gondii* infection could not be detected in any of the cases using single tube nested PCR. In one hydrosalpinx case, *Corynebacterium ovis* (or *C*. *pseudotuberculosis*) was detected by PCR using specific primers. The results for PCR tests are shown in Table 2.

**Table-2:** Results of PCR tests employed on tissue specimens of reproductive organs of female goats.

Pathogenic organisms	Total number of cases selected for PCR	Number of positive cases by PCR	Incidence out of total PCR n=76 (%)	Incidence out of total samples collected n=660 (%)	Incidence out of total pathological lesions n=154 (%)
*B. melitensis*	N=76	12	15.78	1.81	7.79
*Chlamydia* spp.		3	3.94	0.45	1.94
*Campylobacter* spp.		35	46.05	5.30	22.72
*T. gondii*		0	0	0	0
*C. ovis* (*pseudotuberculosis*)		1	1.31	0.15	0.64
Total	76	51	67.10	7.72	33.11

*B. melitensis=Brucella melitensis, T. gondii=Toxoplasma gondii, C. ovis=Corynebacterium ovis,* PCR=Polymerase chain reaction

**Figure-1:**
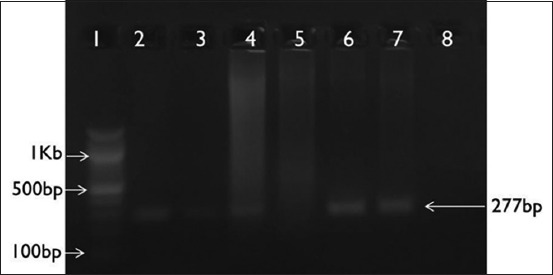
*Chlamydophila* spp. *16srRNA* gene-based polymerase chain reaction. Lane 1: 100 bp ladder; Lane 2: Positive control; Lane 3-7: Unknown samples; Lane 8: No template control.

**Figure-2:**
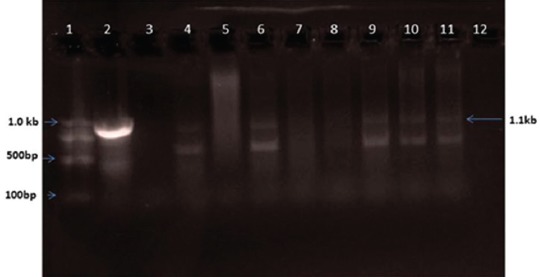
*Brucella melitensis* OMP31 gene-based polymerase chain reaction. Lane 1: 100 bp ladder; Lane 2: Positive control; Lane 3-11: Unknown samples; Lane 12: No template control.

**Figure-3:**
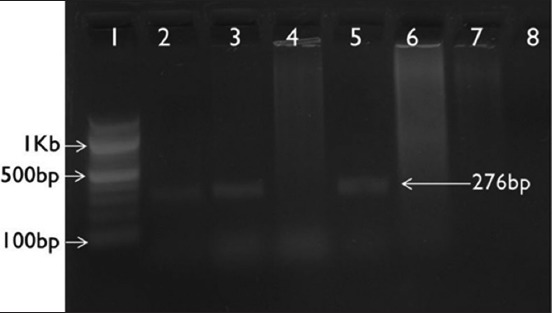
*Campylobacter* spp. *16srRNA* gene-based polymerase chain reaction. Lane 1: 100 bp ladder; Lane 2: Positive control; Lane 3-7: Unknown samples; Lane 8: No template control.

**Figure-4:**
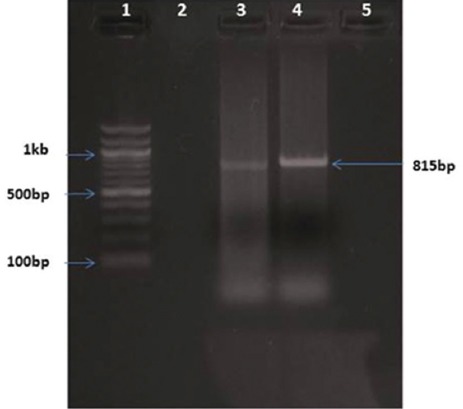
*Corynebacterium ovis 16srRNA* gene-based polymerase chain reaction. Lane 1: 100 bp ladder; Lane 2: Positive control; Lane 3: Unknown samples; Lane 4: No template control.

Consequent on PCR amplification and agarose gel electrophoresis of the DNA extracted from tissue samples, a 277 bp amplicon was observed in *Chlamydia*-positive samples (Figure-1), in *B. melitensis-*positive samples, 1.1 kb size amplicon (Figure-2), in *Campylobacter*-positive samples, 276 bp amplicon (Figure-3), and in *C. ovis*-positive samples, 815 bp amplicon (Figure-4) was observed.

**Figure-5:**
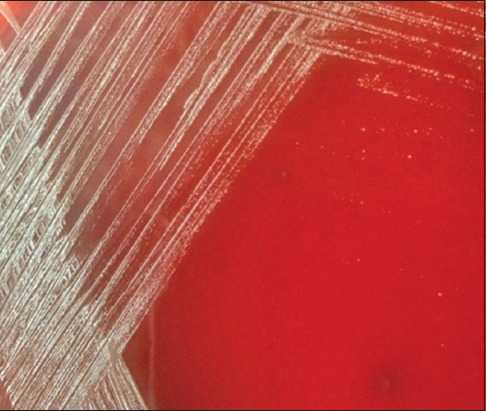
*Brucella* colonies were pearly white, with smooth margins, translucent in brucella blood agar.

**Figure-6:**
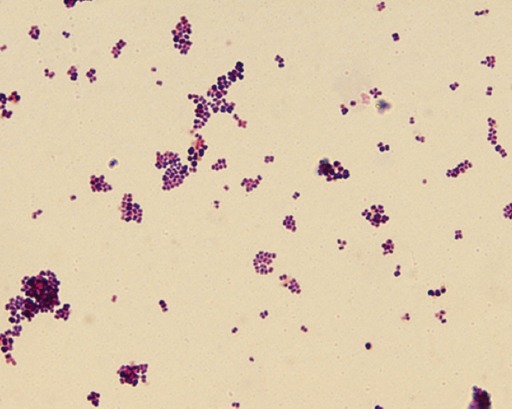
Brucella organisms were seen as coccobacilli/pleomorphic short rods, arranged singly or in pairs or in small groups (Gram’s stain, 1000×).

In the present investigation, *B. melitensis* infection was detected in 12 (1.81%) cases, which was confirmed by species-specific (OMP-31) PCR as well as by bacterial isolation in *Brucella* agar media containing 5% defibrinated sheep blood. After 4-day incubation, *Brucella* growth colonies characterized by round, 1-2 mm in diameter, convex and pearly white, with smooth margins, translucent, and pale honey-colored colonies were observed (Figure-5). Later, the colonies became larger and slightly darker. On microscopic examination of stained slides of colonies in oil immersion (100×), the organisms were seen as Gram-negative coccobacilli or pleomorphic short rods, arranged singly or in pairs or in small groups (Figure-6).

**Figure-7:**
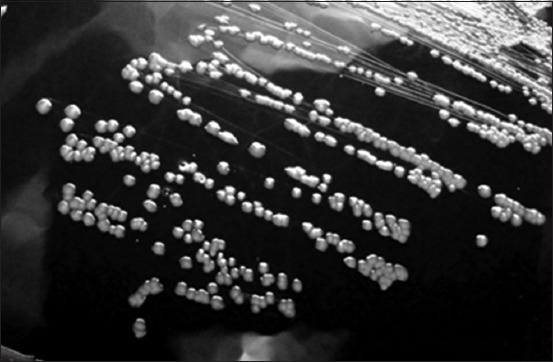
*Corynebacterium ovis*: Circular, convex, and grayish colonies on blood agar.

**Figure-8:**
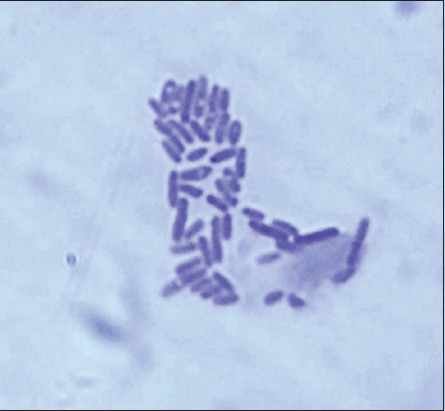
*Corynebacterium ovis*: Gram-positive pleomorphic rods arranged like “Chinese letters” (Gram’s stain, 1000×).

Of 2 (1.29%) cases of hydrosalpinx, one showed the presence of *C. ovis*, and it was isolated in 5% sheep blood agar, and stained bacterial growth colonies showed the presence of Gram-positive bacilli arranged as Chinese letters or in “palisade” arrangement (Figures-7 and 8).

**Figure-9:**
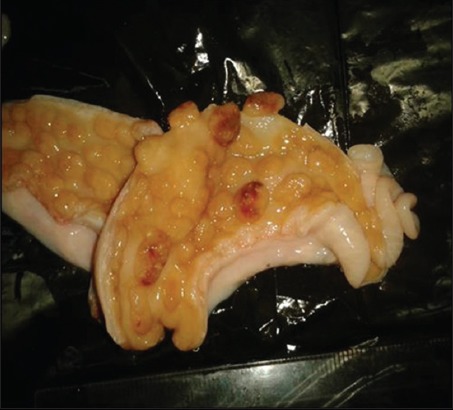
Uterus: Caruncular tips showing necrotic foci and petechial hemorrhages.

**Figure-10:**
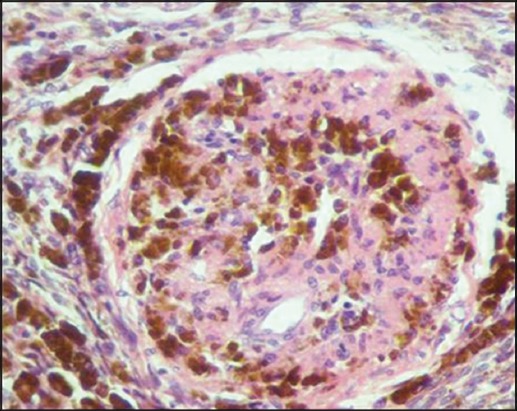
Uterus: Hemosiderin deposition in the caruncular area (hematoxylin and eosin, 400×).

**Figure-11:**
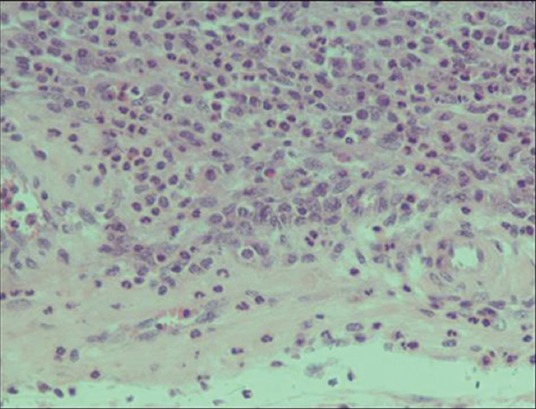
Acute endometritis: Infiltration of neutrophils, lymphocytes, and plasma cells (hematoxylin and eosin, 400×).

**Figure-12:**
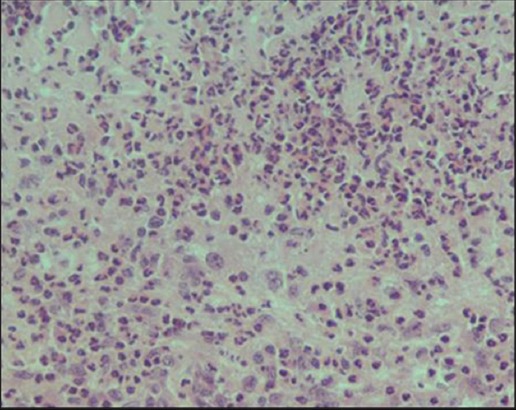
Chronic purulent endometritis: Note extensive neutrophilic infiltration, admixed with macrophages and lymphocytes, and occasionally foreign-body type giant cells (hematoxylin and eosin, 400×).

Grossly, the major pathological lesions noticed in the uterus were petechial hemorrhages over caruncular tips with small, raised, whitish foci scattered in the area (Figure-9). Microscopically, tissue sections showed the presence of hemosiderin deposition with mild-to-severe cellular reactions, comprising predominantly lymphocytes and macrophages (Figure-10). Majority of such of lesions were seen in association with *Campylobacter* infection confirmed by 16srRNA PCR. Acute endometritis lesions were microscopically characterized by mucosal and submucosal infiltration of neutrophils in wide areas admixed with lymphocytes and plasma cells and cellular debris associated with multifocal erosions of the superficial epithelium (Figure-11). Chronic purulent endometritis was histologically characterized by the presence of large central core area of organized pus with abundant neutrophils, irregular calcification areas surrounded by neutrophils predominantly admixed with macrophages and lymphocytes and occasionally foreign-body type giant cells and fibrous encapsulation (Figure-12). The mucosal and submucosal area got infiltrated with lymphocytes; chronic endometritis with fibroplasia and atrophy of glands were noticed. In this sample, *Campylobacter* infection was confirmed by PCR.

**Figure-13:**
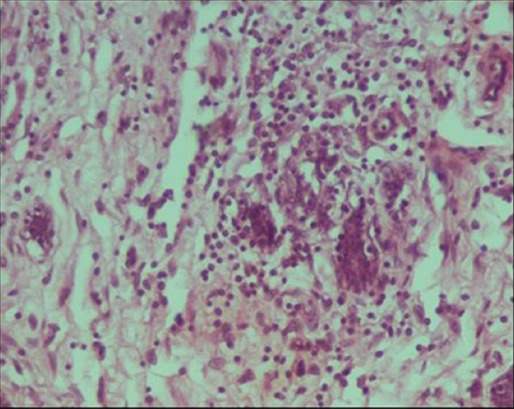
Chronic nonpurulent endometritis: Focal infiltration of lymphocytes, plasma cells, macrophages, and fibroblasts (hematoxylin and eosin, 400×).

**Figure-14:**
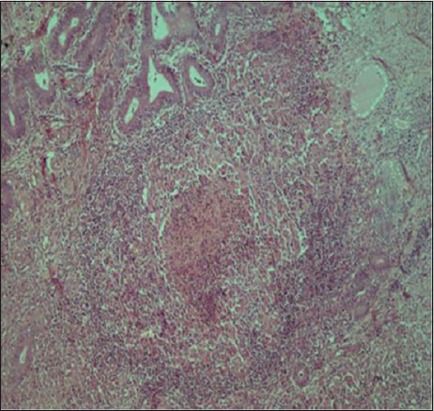
Chronic granulomatous endometritis: A discrete microgranuloma in uterine wall (hematoxylin and eosin, 400×).

**Figure-15:**
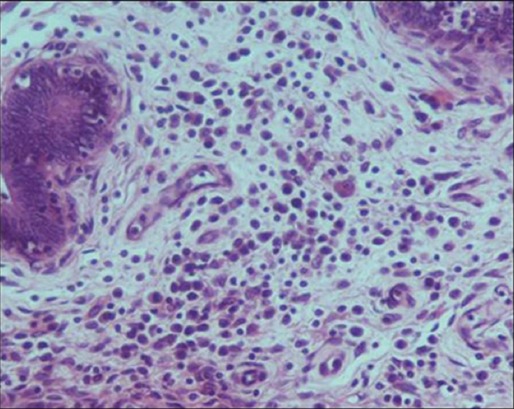
Chronic granulomatous endometritis: Mononuclear cells, lymphocytes, macrophages, epithelioid cells, and plasma cells in the submucosal area (hematoxylin and eosin, 400×).

Chronic nonpurulent endometritis was histopathologically characterized by the presence of diffuse or focal lymphocytic infiltration, macrophages, plasma cells and fibroblast in the lamina propria, and multifocal desquamation of surface epithelium leaving behind denuded basement membrane. The epithelial lining of endometrium was partly or completely sloughed and necrosed (Figure-13). Chronic granulomatous endometritis was noticed in two cases exhibiting the presence of micro granulomas (Figure-14). *B. melitensis* was isolated from these cases and was also confirmed by PCR using *OMP 31* gene species-specific primers. In *B. melitensis* infected cases, the major uterine histopathological change was chronic endometritis characterized by infiltration of mainly mononuclear cells, lymphocytes, macrophages, epithelioid cells, and plasma cells in the mucosal and submucosal areas (Figure-15).

**Figure-16:**
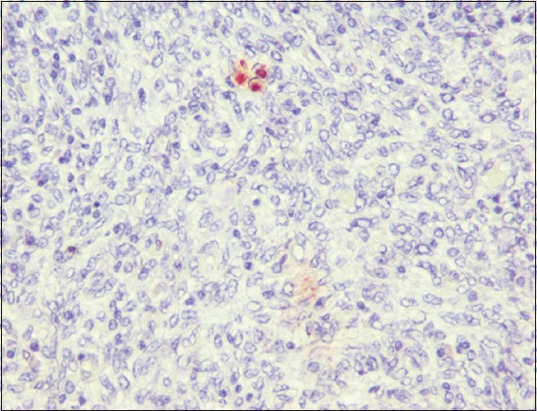
Uterus: *Brucella melitensis*-positive cytoplasmic immunolabeling of macrophages (IPO-3-amino-9-ethylcarbazol-Mayer’s hematoxylin counterstain, 400×).

The tissue sections used for IHC in the present study were from samples found positive by PCR for *B. melitensis*. Positively stained tissue sections evinced foci of cells with cytoplasmic immunolabeling of macrophages indicating the presence of *B. melitensis* antigens/organisms in abundance, pushing the nuclei on the periphery (Figure-16).

Ovarian lesions occurred in frequency next to the uterine pathologies and included ovario-bursal adhesions, paraovarian cysts, polycystic ovary, cystic follicles, luteal cysts, ovarian tumor, ovarian atrophy, oophoritis, congestion, and hemorrhages. Ovaro-bursal adhesions were mostly seen in association with oophoritis. PCR test of affected tissues revealed the presence of potent pathogens such as *Brucella* and *Campylobacter*.

## Discussion

Uterine infections not only disrupt the function of the uterus but also the ovary and the control centers in the hypothalamus and pituitary gland. Most uterine infections begin in the endometrium and are associated with mating, pregnancy, or postpartum uterine involution. Therefore, proper diagnosis and treatment of uterine diseases were considered as a key component of all reproduction management programs. In California, a survey revealed that 30% of uterine infections leading to abortion in goats were caused by bacteria, 4% by protozoa, and remaining by virus and fungi [19]. The presence of potentially pathogenic bacteria in the female genital tract might adversely affect the reproductive performance of sheep and goats [20].

The overall incidence of pathological lesions in the female genitalia of goats was found to be 16.3% in this study. This incidence was considered higher in comparison to the findings of Francis [7] and Archana *et al*. [21] who recorded incidences ranging from 1.9% to 21.2%. The variation in the incidence of pathological lesions in the present study compared to that of other workers from India or abroad might be due to differences in the breed of the animal, managemental practices, and climatic/geographical conditions.

The pathological conditions of the uterus were highest in the current study with an overall incidence of 12.42% [22]. Regularly observed gross lesion in the uterus was petechial hemorrhages on the tips of uterine caruncles. In majority of these cases, *Campylobacter* spp. infection was detected. An experimental study conducted by Hedstrom *et al.*, in ewes, showed that gross lesions were present in caruncles of all *Campylobacter jejuni* inoculated ewes and revealed severe vasculitis and endometritis [23]. These findings corroborated with ours in which most of the uterine samples grossly exhibited hemorrhagic tips of caruncles which were positive for *Campylobacter* spp. infection by PCR.

In some cases, the lumen of uterine glands contained inflammatory cells along with the secretion. In *Chlamydia* infections, earlier workers observed the presence of neutrophils in uterine endometrial and submucosal glandular lumen in humans and guinea pigs, respectively [24,25]. Endometritis may follow certain conditions such as dystocia, fetal maceration, or retention of fetal membranes [26]. Pathogenic organisms such as *Brucella* and *Chlamydia* were isolated from acute endometritis cases of this study. Lymphoplasmacytic endometritis may be associated with persistent infection of *Chlamydophila* spp. [27].

In the present study, all the three positive cases of *Chlamydia* infection were seen in association with placental retention. Placental retention was frequently seen in chlamydial abortions of goats compared to sheep [28]. The presence of chlamydial antigens in the vagina, uterus, and uterine tubes after a year of abortion was confirmed by PCR and bolstered by demonstration of antigens in the uterus and fallopian tube using IHC [27]. However, we could not attempt IHC for *Chlamydia* infection presently. Rahman [29] from Bangladesh unsuccessfully attempted for isolation and identification of *Chlamydiae* from the reproductive tract of slaughtered does. Even though 9 species were present, *Chlamydophila abortus* was recognized as common etiological agent involved in small ruminant abortion [30]. In Hungary, during a study period of 7.5 years, it was found that the main cause for ovine and caprine abortions were *Chlamydophila abortus* infection with a prevalence of 46% and 17%, respectively [31]. PCR proven to be a sensitive and reliable test has become an important diagnostic method for *Chlamydia* infection in animals [32,33]. The *Chlamydiae* are normally shed more abundantly in estrus than during the diestrus period [34].

*Campylobacter* spp. was detected in one particular case of purulent chronic endometritis by PCR. Rahman *et al*. [35] found that severe uterine infection causes subserosal abscess. Hence, it is assumed that the persistent *Campylobacter* infection leading to prolonged inflammatory reaction resulted in the development of myometrial abscess. *Campylobacter* spp., mainly *C. jejuni* and *C. coli* are the important etiological agents causing abortion as well as severe gastroenteritis in humans. Among slaughtered goat meat, there is 73% contamination by *Campylobacter coli* and 12% by *C. jejuni* infection. The studies in Eastern Congo, Ethiopia, and Nigeria revealed that *Campylobacter* in slaughtered animals occurs due to poor handling of carcass and cross contamination [36]. Overall, the prevalence of 34.6% of *Campylobacter* spp. was detected in goats slaughtered at Congo and in developing countries such as Iran, Pakistan, and Malaysia, and *Campylobacter* was reported in 9.4% of goat meat [37].

Toxoplasmosis is a widespread zoonotic disease caused by *T. gondii* characterized by abortions, stillbirth, mummification, and congenital anomalies depending on the stage of gestation in does as well as in humans. Sharma *et al*. [38] sited a low seroprevalence of *T. gondii* among the ruminants in India. They found antibodies for *T. gondii* in 7 of 186 (3.763%) sheep, 2 of 83 (2.409%) cattle, and 3 of 103 (2.913%) buffaloes. These results indicated a low prevalence of *T. gondii* in ruminants tested. In Kerala, a total of 98 goats screened for the presence of toxoplasma antibodies, and 58 were found positive [39]. Among 13 seropositive goats, the uterus of one goat showed the presence of the parasite DNA by PCR in Brazil [40]. In the present study, all samples were negative for *T. gondii* by PCR.

*B. melitensis*, the etiological agent of brucellosis in goats, is a zoonotic infection and is most pathogenic for humans. Higher seroprevalence was recorded in female goats compared to male [41,42], providing a potential reservoir for the organism to propagate. A report from Egypt revealed that in each year the incidence of *Brucella* infection among slaughtered goats was found to be more compared to other species such as buffalo, sheep, and cattle [43]. These studies have confirmed that brucellosis is still endemic among the slaughtered animals in the study area and the prevalence is increased, especially among the small ruminants. In India, a few studies have reported the seroprevalence of brucellosis in goats of different states [44]. The cumulative incidence of brucellosis in sheep and goat was found to be 7.9% and 2.2%, respectively [45]. However, there is no traceable report about the incidence of *B. melitensis* in genital tract of does. PCR test for *Brucella* using tissue samples was found to be 86% sensitive and 100% specific [46].

IHC on formalin-fixed paraffin-embedded tissues is a rapid, specific, useful technique for the diagnosis of *B. melitensis* infection [13]. In the present study, immunohistochemical staining was performed using standard ABC method for the detection of *B. melitensis* in the genitalia. Using this technique, the organism in the uterus could be located in the cytoplasm of inflammatory cells, especially the macrophages of fixed tissues [47]. These findings show the role of macrophages in *Brucella* infection. A study conducted previously in bucks infected with *B. melitensis* found that there was a positive correlation between the distribution of *B. melitensis* and IHC intensity [48]. In this study, out of total, 12 positive cases confirmed by species-specific (0MP31) PCR, 6 cases were subjected to IHC, and only 3 cases showed positive immunolabeling.

Bilateral hydrosalpinx causes sterility, but unilateral condition causes varying degrees of infertility in goats [7]. Hydrosalpinx occurs as a result of irreversible stage of inflammatory conditions and can make the animal sterile [2]. Earlier studies of hydrosalpinx in cattle revealed that the most prevalent bacteria recovered from hydrosalpinx conditions were *Corynebacterium* and *Actinomyces* with an incidence of 42.8% and 28.6%, respectively [1]. Hence, it was confirmed that there was a correlation between bacterial isolates and hydrosalpinx. These organisms form emboli with consequent infarction and scarification, which will eventually block the lumen of the genital tract. Dawood reported a case of hydrosalpinx in infertile goats associated with cystic lesions in the genitalia [49]. In humans and animals, hydrosalpinx causes a negative effect on gametes and fertilization thereby lowering the endometrial receptivity and results in impairment or reduction in pregnancy rate [50].

In this study, ovarian lesions were seen as the second most prevalent lesion next to uterine pathologies [22]. The study revealed the presence of potent pathogens such as *Brucella* and *Campylobacter* causing inflammation and fibrinopurulent peritonitis which resulted in adhesion of ovary with bursa. Peritonitis or endometritis causes adhesion of ovary with either bursa or with salpinx in goats [7].

## Conclusion

It is concluded from the study that different offending pathogens such as *Brucella*, *Chlamydia*, *Corynebacterium*, and *Campylobacter* were involved in causing various pathological conditions of female reproductive organs of goats. Most of the infectious reproductive problems in animals remain unnoticed until some clinical signs such as abortion or stillbirth develop. The best hope for reducing the infertility and sterility in animals depends on the early diagnosis of such pathogens that lead to infertility. In most of the cases, brucellosis and chlamydial infections could be difficult to differentiate; however, a battery of tests may be employed to facilitate their diagnosis including histopathology, PCR, and IHC for brucellosis. Goats attain early sexual maturity with regular and successful reproduction and produce about 2 kids/year. However, such prolificacy often fails to be attained on account of pathological problems related to genital tract in female goats. Most conventional farms only employ *Brucella* screening, but with this study, it is evident that the reproductive diseases and infertility are caused by other organisms including campylobacteriosis, chlamydia, and even corynebacterial infections. Hence, it is paramount to address the reproductive problem with broad unbiased diagnostic methods.

## Authors’ Contributions

VB, RVSP, and KG designed and conducted the research work. DDS, NKG, and AKM collected and analyzed the samples; VKG, RS, AKS, MK, and AK helped in preparing and reviewing the manuscript. All authors read and approved the final manuscript.
